# PiNN: Equivariant Neural Network Suite for Modeling Electrochemical Systems

**DOI:** 10.1021/acs.jctc.4c01570

**Published:** 2025-01-30

**Authors:** Jichen Li, Lisanne Knijff, Zhan-Yun Zhang, Linnéa Andersson, Chao Zhang

**Affiliations:** † Department of Chemistry-Ångström Laboratory, Uppsala University, Lägerhyddsvägen 1, P.O. Box 538, 75121 Uppsala, Sweden; ‡ Wallenberg Initiative Materials Science for Sustainability, Uppsala University, 75121 Uppsala, Sweden

## Abstract

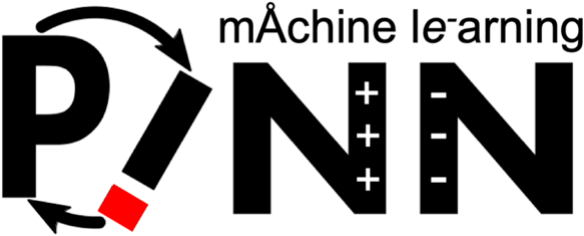

Electrochemical energy storage and conversion play increasingly important roles in electrification and sustainable development across the globe. A key challenge therein is to understand, control, and design electrochemical energy materials with atomistic precision. This requires inputs from molecular modeling powered by machine learning (ML) techniques. In this work, we have upgraded our pairwise interaction neural network Python package PiNN via introducing equivariant features to the PiNet2 architecture for fitting potential energy surfaces along with PiNet2-dipole for dipole and charge predictions as well as PiNet2-χ for generating atom-condensed charge response kernels. By benchmarking publicly accessible data sets of small molecules, crystalline materials, and liquid electrolytes, we found that the equivariant PiNet2 shows significant improvements over the original PiNet architecture and provides a state-of-the-art overall performance. Furthermore, leveraging on plug-ins such as PiNNAcLe for an adaptive learn-on-the-fly workflow in generating ML potentials and PiNNwall for modeling heterogeneous electrodes under external bias, we expect PiNN to serve as a versatile and high-performing ML-accelerated platform for molecular modeling of electrochemical systems.

## 1 Introduction

In the United Nations’ resolution “Transforming our world: the 2030 Agenda for Sustainable Development” published in 2015, 17 sustainable development goals (SDGs) were outlined. In the same year, The Paris Agreement states that the rise in the global mean temperature should be kept preferably within 1.5 °C. To achieve these goals in a cost-effective way and to address societal challenges across the globe, electrochemistry and electrochemical energy materials play a pivotal role.^1,2^

One of the grand challenges in electrochemistry is to understand, control, and design electrochemical energy materials at atomistic precision. It requires establishing quantitative relationships between the macroscopic properties such as current and voltage and the time evolution of the corresponding bulk materials, interfaces, and interphases at the microscopic scale. This crucial information can be very difficult to extract from the top-down approach commonly used in experiments. Therefore, molecular modeling based on quantum mechanical and statistical mechanical principles becomes indispensable.^3^

AI and machine learning (ML) have been the tour de force in the field of molecular modeling. The 2024 Nobel Prize in Chemistry given to computational protein design and structure prediction is an appreciation of this breakthrough. For materials systems,^4^ ML has been used to accelerate molecular simulation with machine learning potentials (MLPs) which provide quantum mechanical accuracy at a fraction of the cost,^5−7^ to identify reaction coordinates automatically for enhanced sampling and free energy calculations,^8−10^ and to propose new structures with generative AI for inverse design,^11−13^ among many other exciting and emerging applications.^14−16^

A notable advancement in the development of MLPs was the introduction of equivariant features into graph convolutional neural networks (GCNNs) around 2021. The NequIP architecture^17^ is a pioneer in this advancement among many other equivariant GCNNs such as PaiNN,^18^ EGNN,^19^ and NewtonNet,^20^ that appeared in the same period. It has been shown that GCNNs with equivariant features can lead to much better force and energy predictions and become more data efficient. This motivates us to incorporate equivariant features into our invariant GCNN architecture PiNet, which was first introduced and implemented in our pairwise interaction neural network package PiNN.^21^

Compared to general-purpose packages such as DeePMD^22^ and MACE,^23^ our development of PiNN has its purpose for modeling electrochemical systems from the beginning. This requires taking into consideration not only the accuracy of the Hamiltonian and the precision of sampling but also the electric boundary conditions. Keeping that in mind, we have developed special-purpose modules such as PiNet-dipole^24^ for predicting dipole and charge and PiNet-χ^25^ for predicting polarizabilty and generating a charge response kernel under external bias. These have further led to the birth of PiNNwall^26^ that joins forces from the PiNN and MetalWalls codes for modeling heterogeneous electrodes in fully solvated electrochemical environments.

In this work, we introduced equivariant features (P3 and P5) into the second generation of PiNet. PiNet2-P3 shows a significant and cost-effective improvement on energy and force predictions across different types of data sets ranging from small molecules to liquid electrolytes, as compared to PiNet. The equivariant features turn out to also significantly improve the dipole and polarizability predictions, as demonstrated by the upgraded PiNet2-dipole and PiNet2-χ. In the following, we first introduce the new implementations of equivariant features in PiNet2 and special-purpose modules PiNet2-dipole and PiNet2-χ. This is followed by the introduction of plug-ins PiNNAcLe for an adaptive learn-on-the-fly workflow and PiNNwall for molecular simulations of electrode/electrolyte interfaces. Then, comparisons of performance between PiNet and PiNet2 on different public data sets including small molecules, crystalline materials, and liquids will be presented. Finally, the applications of PiNN to two realistic electrochemical systems are demonstrated.

## 2 Architectures and Modules in PiNN

### 2.1 Equivariant Neural Network PiNet2

In the original PiNet, we reformulated the GCNN as pairwise interaction (PI) operations and interaction pooling (IP) operations. After PI and IP operations, the atomic and pairwise information is updated the same way as a message-passing graph convolution block. The initial and invariant (*n* = 1) atomic property  is generated using either the nuclear charge *Z*_*i*_ or one-hot embedding. The invariant (*n* = 1) pairwise interaction  is a function of the initial atomic properties of two atoms and their distance *r*_*ij*_
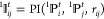
1where *t* is an iterator and *t* + 1 goes up to the number of graph convolution (GC) blocks. This is followed by the IP operation, where summation ensures the permutation invariance of the generated atomic property
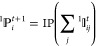
2

The PI + IP abstractions of GCNN and the unique design of a weight matrix *W*_*ij*_ using information from both  and  in the PI operation as  are the core of PiNet, where NN stands for a standard feedforward neural network (NN). More details about the architecture of PiNet, for instance, the generation of the radial basis function *e*_*ij*_ using the information on *r*_*ij*_, can be found in the previous PiNN publication^21^ and the online documentation of PiNet https://teoroo-cmc.github.io/PiNN/master/usage/pinet/.

In PiNet2, the vectorial atomic property, , contains an additional dimension *x* accounting for directional information. Accordingly, the vectorial pairwise interaction  does not only include the pairwise distance but also the bond vector ^3^*r*_*ijx*_
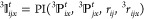
3

In the case of the IP operation, the same summation in eq 2 applies to the vectorial pairwise interaction . However, the corresponding operation for the invariant atomic properties is modified as
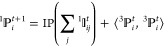
4

**Figure 1:**
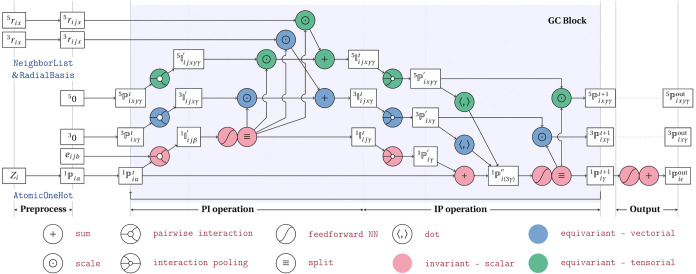
Architecture of equivariant neural network PiNet2. The original invariant PiNet architecture shown at the bottom (colored in red) provides the base for PiNet2. PiNet2-P3 incorporates additional vectorial features (colored in blue) and PiNet2-P5 includes further tensorial features (colored in green). The GC block is highlighted with a purple background. Note that Greek letters such as α, β, and γ indicate the number of channels in the corresponding feature vector. They should not be confused with atomic indices *i* and *j* and directional components *x* and *y*. Prime and double-prime are intermediate objects during the PI and IP operations. See the online documentation https://teoroo-cmc.github.io/PiNN/master/usage/pinet2/ for detailed explanations of the network structure and implementation of different actions (such as “scale” and “dot” for equivariant features).

The overview of the PiNet2-P3 architecture is shown in the middle part of Figure 1.

Similarly, one can add higher-order equivariant features to the network. For example, the tensorial atomic property  incorporating ^5^*r*_*ijxy*_ and its corresponding PI and IP operations have also been implemented in PiNet2 (see the top part of Figure 1 colored in green). Here, ^5^*r*_*ij*_ is the five-component irreducible representation of the outer product of the bond vector, which was used in practice for computational efficiency.

### 2.2 Predicting Dipole and Charge with PiNet2-Dipole

The PiNet-dipole model was designed to predict the dipole moment of both molecules and condensed-phase systems.^24^ The original model implemented in the PiNet-dipole is here referred to as the atomic charge (AC) model. The AC model is based on the following equation
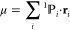
5

Here, **r**_*i*_ is the position of atom *i*.  is the model prediction which can be interpreted as an atomic charge. To enforce a total system charge of zero, a charge neutrality constraint is enforced. When PiNet2 is used as the back-end,  is still a scalar but contains information from the higher-order geometrical features via eq 4.

The introduction of vectorial features opens up alternatives for dipole moment prediction. Therefore, PiNet2-dipole is an assembly of methods rather than a specific model. The most straightforward way to utilize vectorial features is through the summation of  features, in the atomic dipole (AD) model
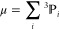
6

 can be seen as an atomic dipole moment in this case, and is only accessible in PiNet2.

Finally, the IP and PI formulation in GCNN in PiNN can be exploited to predict the dipole moment through the following equation
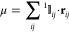
7where **r**_*ij*_ is the bond vector and  is the pairwise interaction mentioned before (eq 1). As the  may be interpreted as a bond charge between a pair of atoms, this type of model is referred to as the bond charge (BC) model.

Besides combining these base models, we also developed other variants on top of them. One is applying an L2-regularization (R) term to shrink the predictions as small as possible. The other is the inclusion of an oxidation state (OS) term when the dipole moment is predicted. This means the AD(OS) model is defined as
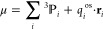
8

Here, the second term is the OS contribution, which contains the formal oxidation state *q*_*i*_^os^ of each atom *i*.

### 2.3 Predicting Molecular Polarizability with PiNet2-χ

PiNet-χ provides an assembly of ML models that we developed previously to generate the atom-condensed charge response kernel (CRK) by regressing the molecular polarizability.^25^ The CRK χ describes the response charge from an external potential based on density functional linear response theory^27^ and its atom-condensed form is related to the polarizability tensor **α** as^28^
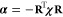
9where **R** is an *N* × 3 matrix of atomic coordinates.

We have used different ansatz from chemistry and physics to bring physical constraints to the ML-constructed χ in addition to the basic requirements from the symmetry and sum rule. The CRK **χ** is related to the hardness kernel **η** and the softness kernel **η**^–1^ through the relation^29^
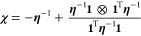
10

The electronegativity equalization (EEM)-type model and the EtaInv-type model were developed based on this relation. In the case of the EEM-type model, the atomic property  was used to predict the environment-dependent on-site parameters η_*ii*_; in the case of the EtaInv-type model, the atomic property  and the pairwise interaction  were combined to directly predict the softness kernel **η**^–1^.

The other ansatz that we explored previously is Dyson’s equation
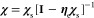
11where **χ**_s_ is the irreducible response kernel for the noninteracting system. We proved in our work^25^ that Dyson’s equation is equivalent to the atom-condensed Kohn–Sham approximated to second-order (ACKS2) method.^30^ In the ACKS2-type model, atomic property  was used to predict **η**_e_ and the symmetrized pairwise interaction  was used to predict **χ**_s_.

The local ansatz was also explored previously in the Local-type model, where the total χ is written as the sum of atomic contribution χ_*i*_ and χ_*i*_ was constructed using the tensor product of pairwise interaction . In PiNet2-χ, all flavors including the EEM-type, the EtaInv-type, the ACKS2-type, and the Local-type have been upgraded thanks to the inclusion of vectorial features  into  and  via eqs 4 and 1, respectively.

## 3 Available Plug-Ins for PiNN

### 3.1 Adaptive Learn-on-the-Fly Plug-In PiNNAcLe for Generating Machine Learning Potentials

Applications of MLPs are often limited by the generalizability thereof, and the difficulty of controlling it, especially when the simulation time scale goes beyond what is possible for the reference calculations. An adaptive learn-on-the-fly (AcLe) algorithm has been developed to address this challenge, along with workflows PiNNAcLe that interface PiNN with different packages that perform electronic structure calculations, such as CP2K.^31,32^

In a nutshell, the AcLe algorithm performs the validation (labeling) of an MLP at an adaptive time scale instead of relying on the uncertainty quantification methods that assume the correlation between the test error and the estimated error. This provides an alternative as compared to popular active learning schemes.^33−37^ This makes PiNNAcLe particularly useful for predicting properties that require robust sampling at a much longer time scale, such as the computation of transport coefficients in concentrated electrolyte solutions using linear response theory.^38^

Details of the AcLe algorithm and the corresponding code PiNNAcLe are described and distributed elsewhere.^39^

### 3.2 Heterogeneous Electrode Plug-In PiNNwall for Molecular Simulation of Electrode–Electrolyte Interfaces

The PiNNwall plug-in allows for PiNN to interface with the MetalWalls code^40,41^ and to perform molecular dynamics simulations of electrochemical systems that involve heterogeneous electrodes, which goes beyond what the standard Siepmann–Sprik model^42^ can offer. Here, heterogeneous electrodes refer to the ones that contain multiple chemical elements, atomic defects, surface corrugation, or functionalization.^43^

The PiNNwall interface reads in the electrode structure and then predicts the charge response kernel as well as the base charge using PiNet2-χ and PiNet2-dipole, respectively. This information is then passed to MetalWalls to compute the response charge **c** of the electrode atoms under an external field **E**_0_ accordingly
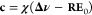
12and to propagate the dynamics of the electrolyte.

Detailed description of PiNNwall and its distribution can be found in our previous publication.^26^

## 4 Case Studies

### 4.1 Crystalline Materials Data Sets: Materials Project

We began our benchmark studies with the MP-crystals-2018.6.1 data set provided by MEGNet,^44^ which includes DFT-computed energies and band gaps for 69,640 crystals from the Materials Project.^45^ We trained on 60,000 crystal structures from this data set, achieving a mean absolute error (MAE) of 29.5 ± 0.5 meV/atom for the prediction of formation energies with PiNet,^21^ compared to MEGNet’s 28 meV/atom with the same training size. The PiNet2-P3 and PiNet2-P5 lead to marginal improvements on this data set, reaching 29 ± 1 and 27.7 ± 0.5 meV/atom, respectively.

The same pattern was observed when we switched to the MPF.2021.2.8 data set provided by M3GNet,^46^ which includes 187,687 ionic steps from 62,783 compounds, corresponding to 187,687 energy values and 16,875,138 force components. As shown in Table 1, the improvement in energy-only training is negligible when switching from invariant PiNet to equivariant PiNet2-P3 and PiNet2-P5. However, much improvement has been seen when training both energy and force simultaneously. PiNet2-P5 leads to an energy MAE of about 34.9 ± 0.7 meV/atom and a force MAE of about 70 ± 2 meV/Å, which can be compared to 34 ± 3 meV/atom and 70 ± 2 meV/Å reported by M3GNet-*EF*.^46^ Nevertheless, it is worth mentioning that both training and inference steps involve a significant computational overhead when switching from PiNet to PiNet2-P5 (see Figure S1 in the Supporting Information) and the most cost-effective improvement is led by PiNet2-P3 instead. Therefore, in the following sections, we focus on the comparison between PiNet and PiNet2-P3. Significant improvements on both energy and force predictions brought in by PiNet2-P3 have been seen for data sets with low symmetry and high disorder (Sections 4.2 and 4.5).

**Table 1:** Mean Absolute Errors (MAEs) of Energy (meV/atom) and Forces (meV/Å) from Invariant PiNet and Equivariant PiNet2-P3 and PiNet2-P5 Trained on the MPF.2021.2.8 Data Set^a^

energy (meV/atom) force (meV/Å)	PiNet	PiNet2-P3	PiNet2-P5
energy	35(4)	35(4)	32.2(4)
	-	-	-
energy & forces	42.0(8)	36(1)	34.9(7)
	89(1)	73(1)	70(2)

a “energy” means the models were only trained on energy, and “energy & forces” means that both energy and forces were used in the training.

### 4.2 Small Molecules Data Sets: QM9 and rMD17

The QM9 and rMD17 data sets are used to benchmark the predictive performance of small molecules. The QM9 data set^47^ contains around 134k small organic molecules with electronic, energetic, and thermodynamic properties computed with the B3LYP functional.^48^ We have used the purified version of QM9 where 3054 molecular structures were excluded from training and evaluation because of inconsistency.^49^ It is found that PiNet2-P3 achieves an MAE of 8.0 ± 0.1 meV in predicting internal energy at 0 K, compared to the 12 meV MAE of the original PiNet for the same data splitting.

The revised MD17 data set^50,51^ consists of molecular dynamics simulations of small organic molecules, providing intramolecular energy and atomic force data. It was derived from the original MD17 data set,^52^ with 100,000 structures whose energies and forces were recalculated with the PBE functional^53^ using tight self-consistent field (SCF) convergence and a dense density functional theory (DFT) integration grid. By incorporation of vectorial features into the network, PiNet2-P3 achieves a significantly higher accuracy for both energy and forces compared to the invariant PiNet (Table 2). This advantage is even bigger when the models were trained on forces only.

**Table 2:** Mean Absolute Errors (MAEs) for Energy and Force from Invariant PiNet and Equivariant PiNet2-P3 Trained on the rMD17 Data Set^a^

	trained on forces only		trained on energy and forces	
energy (meV) force (meV/Å)	PiNet	PiNet2-P3	PiNet	PiNet2-P3
aspirin	-	-	17(3)	5.1(8)
	47.7(6)	11.9(7)	54(3)	17(1)
ethanol	-	-	1.2(2)	1.1(3)
	6.3(8)	5.2(2)	7.8(2)	6.1(9)
malonaldehyde	-	-	3.7(9)	1.4(5)
	14(1)	7.1(5)	14.4(9)	7.5(6)
naphthalene	-	-	8.0(7)	1.4(7)
	31(1)	1.9(1)	33(2)	2.9(1)
salicylic acid	-	-	8.2(6)	2.3(8)
	42(2)	6.1(8)	44(2)	8.3(4)
toluene	-	-	7(2)	1.1(3)
	23(1)	2.2(4)	27.8(4)	3.3(4)
uracil	-	-	7(2)	1.2(3)
	25(2)	3.9(8)	30(3)	5.2(1)

a We used 950 training samples and 50 validation samples in model training. For each molecule and network architecture, three independent models were trained with random data set splits but using the same ratio.

### 4.3 Charge, Dipole, and Polarization Predictions: QM9 and LIQWAT

The benchmarking of the PiNet-dipole models was done on the QM9 data set and a liquid water data set (“LIQWAT”) generated from DFTMD simulations.^24^ For the prediction of charges on this data set, the full vector dipole moment was used to train the models. To compare the CM5 charges,^54^ DFT calculations on the QM9 data set were performed using the same basis set and functional, i.e., B3LYP/6-31G(2df,p), previously.^21^

It is found that PiNet2-dipole outperforms PiNet-dipole in all cases for the QM9 data set with a performance gain of 10% for the AC model as shown in Figure S2 in the Supporting Information. However, this advantage brought by the vectorial features diminishes and becomes marginal when a larger network size was used (Figure S2 in the Supporting Information). When comparing different flavors of models implemented in PiNet2-dipole, AC model shows an MAE of 0.015 D while the AD and AC + BC(R) slightly improve this result with MAEs of 0.014 and 0.013 D, respectively. Therefore, we will focus on the AC model in the following discussion of the QM9 data set.

**Figure 2:**
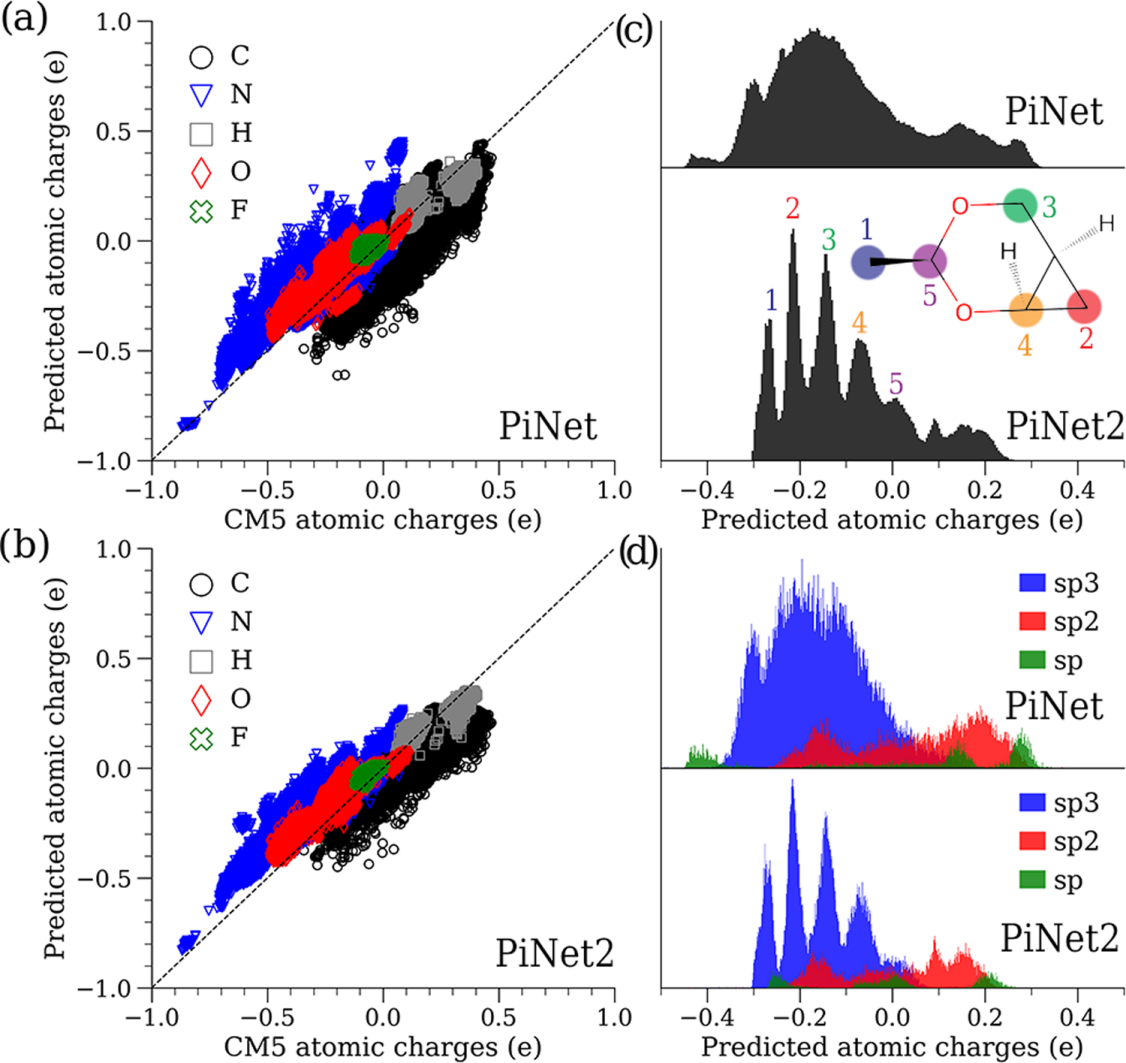
Atomic charges predicted by the PiNet-dipole and PiNet2-dipole models for the QM9 data set. (a) The correlation between the charges predicted by the PiNet-dipole model and the CM5 atomic charges per element. (b) The correlation between the charges predicted by the PiNet2-dipole model and the CM5 atomic charges per element. (c) The distribution of carbon charges predicted by PiNet (top) and PiNet2 (bottom) for the QM9 data set. The different peaks and different chemical environment are illustrated using an exemplar molecule. (d) The distribution of carbon charges predicted by PiNet, and PiNet2 for the QM9 data set separated by hybridization type. The hybridization was determined using the RDKit library.^55^

Figure 2a,b shows a strong correlation between the charges predicted by PiNet-dipole/PiNet2-dipole with the AC model and the charges computed using CM5 population analysis for the QM9 data set. As demonstrated before,^21^ this indicates that the charges from both PiNet-dipole/PiNet2-dipole models have physical meaning because neither models are trained using the CM5 charges as a reference. In addition, the PiNet2-dipole model shows a more narrow spread than the PiNet-dipole model. This means the PiNet2-dipole model predicts charges that are smaller in magnitude than previously, which correspond better with charges derived from CM5 analysis.

When inspecting the predicted charges according to the element types, we found that PiNet2-dipole shows a fine-resolution distribution of sp3 carbon which can not be resolved by PiNet-dipole, as shown in Figure 2c. Taking a molecule from the QM9 data set as an example (Figure 2c), it is clear that these resolved peaks by PiNet2-dipole correspond to different chemical environments for sp3 carbons.

If the carbon atom is surrounded by atoms with a higher electronegativity, its charge becomes more positive. The first peak of the carbon distribution, containing the most electronegative carbons, contains mostly methyl groups. These carbons carry a negative charge as they are bound to the positively charged hydrogen atoms. The carbon atom that is a part of the second peak is a carbon atom bound to two hydrogen atoms and two carbon atoms. This makes it less electronegative than a methyl group as the carbon atoms are less positive than hydrogen atoms, but the carbon is still fairly negatively charged, as true electronegative atoms are absent in its direct chemical environment. When an oxygen atom is introduced, we see that the carbon atom becomes less negatively charged, as indicated by peak 3. This tendency becomes even stronger when the carbon atom is bonded to two electronegative atoms (peak 5). In addition, Figure 2d shows that not only the sp3 distribution of carbon atoms becomes more resolved, but that this effect is also observed for the sp-hybridized carbon atoms.

When it comes to the LIQWAT data set which contains atomic coordinates and supercell dipole moments, the performance increase is approximately a factor of 4 for all of the models when transitioning from PiNet to PiNet2, as shown in Table 3. This is likely to do with the LIQWAT data set, which samples a large conformational space. Table 3 further shows that all models in the PiNet2-dipole family have similarly good performance, except for the BC(R) model.

**Table 3:** Benchmarking Results for Supercell Dipole Prediction from Invariant PiNet-Dipole and Equivariant PiNet2-Dipole Trained on the LIQWAT Data Set^a^

model	MAE (D)	RMSE (D)
PiNet-dipole/AC	0.041(3)	0.062(6)
PiNet-dipole/BC(R)	0.204(1)	0.304(6)
PiNet-dipole/AC + BC(R)	0.047(2)	0.068(3)
PiNet2-dipole/AC	0.008(1)	0.012(1)
PiNet2-dipole/AD	0.010(1)	0.015(1)
PiNet2-dipole/BC(R)	0.048(5)	0.07(1)
PiNet2-dipole/AC+AD	0.010(1)	0.015(1)
PiNet2-dipole/AD(OS)	0.009(1)	0.014(1)
PiNet2-dipole/AC+BC(R)	0.011(1)	0.018(2)

a The descriptions of different models can be found in Section 2.2. The hyperparameters used in the model training are listed in the Supporting Information.

Molecular dipole moment in polar liquids is an interesting quantity to study, because its magnitude and variation are directly linked to the dielectric properties of the polar liquid and to the intensities in infrared spectroscopy. Previously,^24^ it was shown that the molecular dipole moment distribution inferred by the AC model with PiNet-dipole exhibited an excellent correlation with the Wannier dipole resolved from the maximally localized Wannier functions, despite that the AC model was not trained on this at all. This further illustrates the physical soundness of inferring atomic charges from the dipole moment prediction.

**Figure 3:**
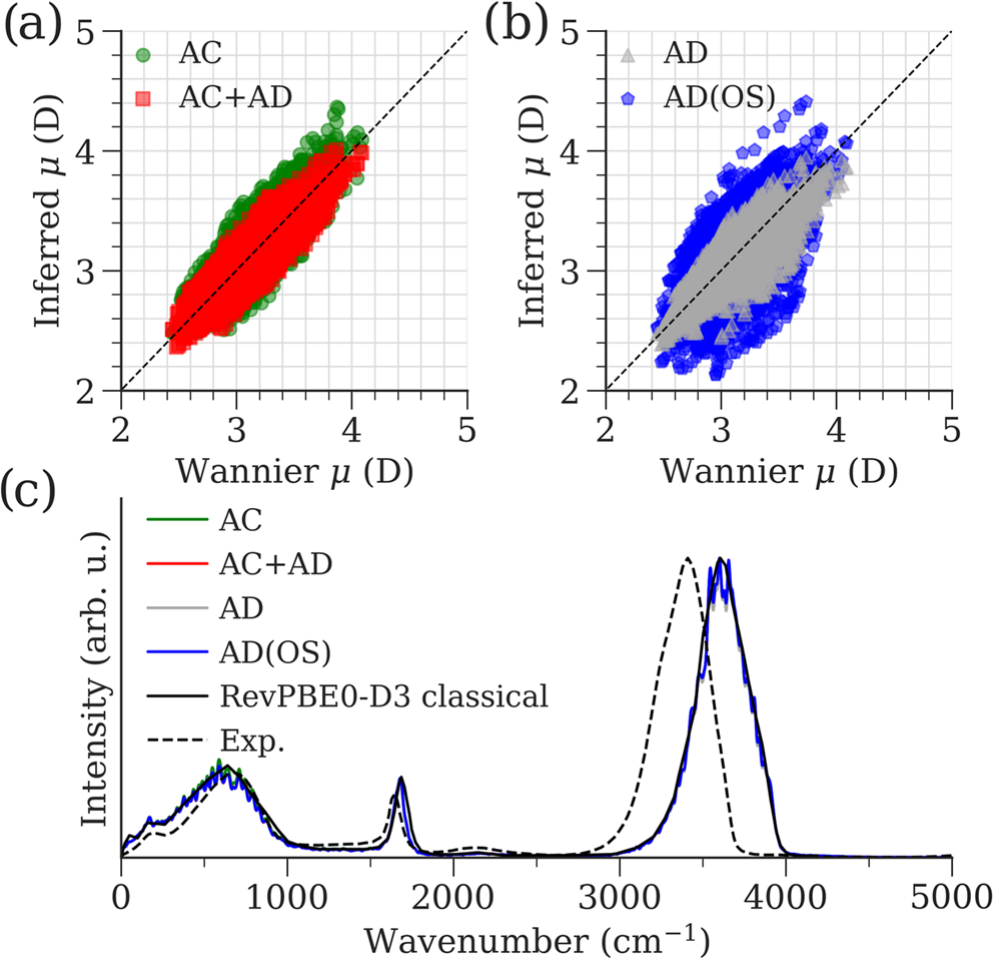
Analysis of the molecular dipole moment and the IR spectra of liquid water using the PiNet2-dipole. (a) Correlation between the molecular dipole moments inferred from the AC and the AC + AD variants of the PiNet2-dipole model with the Wannier molecular dipole moments. The Wannier molecular dipole moments were not used in the training. (b) The same as (a) but for the AD and the AD(OS) variants. See Section 2.2 for the model descriptions. (c) The corresponding IR spectra predicted with PiNet2-dipole. The MLP-based MD simulations were generated using PiNet2-P3/revPBE0-D3 at 300 K and 0.996 g/cm^3^, see Section 4.5 for details. The experimental spectrum^56^ and that from DFTMD simulations (RevPBE0-D3 classical)^57^ are also shown. The difference in the O–H stretching region was attributed to the nuclear quantum effects.^57^

Here, we have repeated this exercise for the PiNet2-dipole as well. Figure 3a shows that the PiNet2-dipole/AC model predicts a molecular dipole moment distribution that corresponds well with the Wannier dipole distribution. This correlation improves when an AD contribution is added to the AC + AD model. The AD and AD(OS) models also show a good correlation with the Wannier center distribution as shown in Figure 3b, but one that is slightly worse than that of the AC-type models. These suggest that a high correlation between the ML-inferred molecule dipole and the Wannier dipole, regardless of the architectures and the models, is not a coincidence.

Finally, we have also applied PiNet2-dipole for computing the IR spectra of liquid water with assistance of the TRAVIS code.^58,59^Figure 3c shows the IR spectra computed for the models discussed in Figure 3a,b, which are nearly identical for all of the models. This is not surprising as the MAEs on predicting the supercell dipole moment from these models are very small and in the order of 0.01 D. The computed IR spectra is virtually identical to that of DFTMD simulations with the same DFT functional,^57^ meaning that that PiNet2-P3 accurately captures the potential energy surface of the system (see Section 4.5), and PiNet2-dipole yields accurate predictions of the corresponding polarization surface.

### 4.4 Polarizability Prediction: QM7b

**Figure 4:**
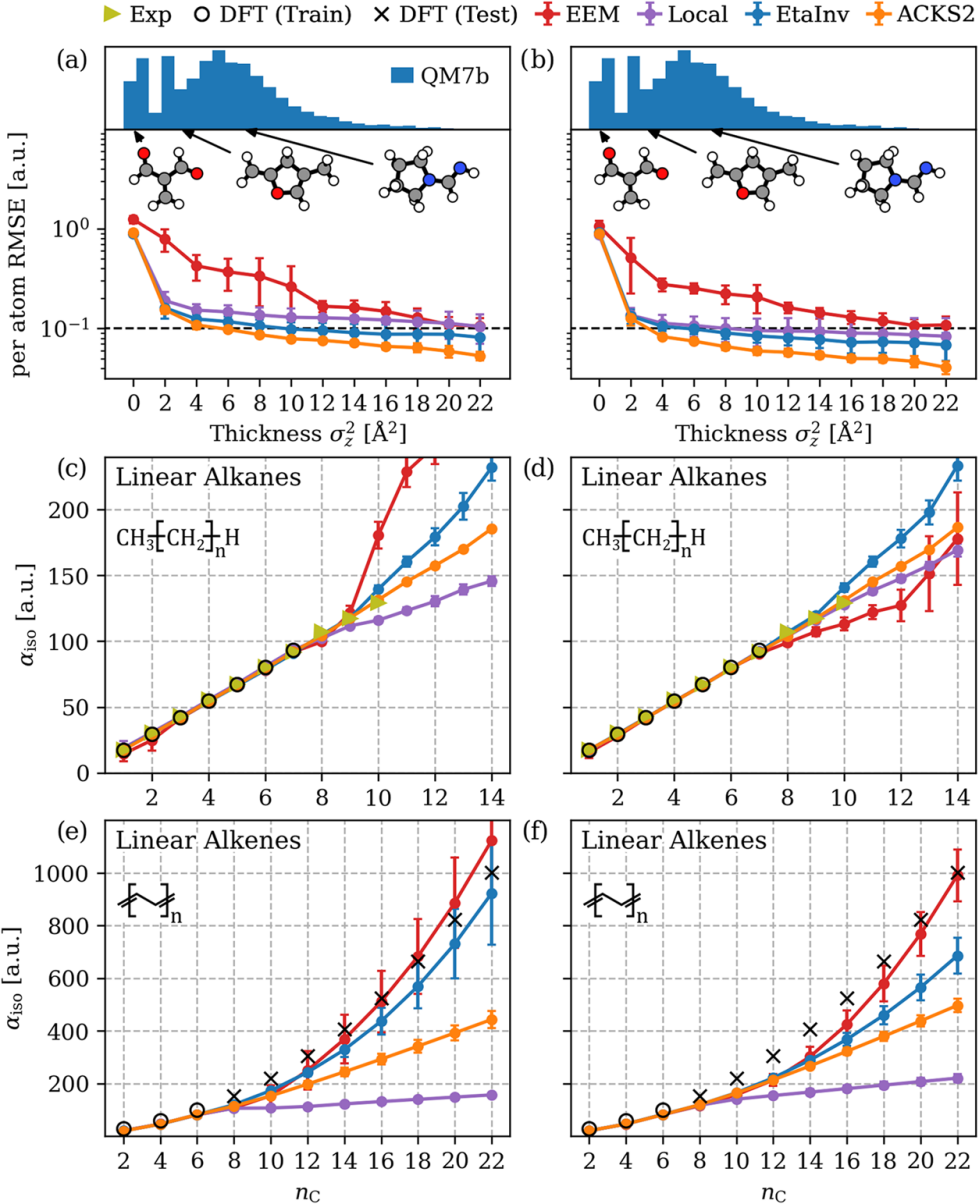
Predicted molecular polarizability from PiNet-χ (a, c, e) and PiNet2-χ (b, d, f) trained on the QM7b data set. The upper panels of (a, b) show the thickness distributions of the entire data set. In the lower panels, the validation RMSE is plotted against the thickness threshold, where molecules with thickness below the given threshold are excluded. (c–f) Scaling of the isotropic polarizability α_iso_ with the number of carbons *n*_C_ in (c, d) *n*-alkanes and (e, f) *n*-alkenes. Experimental values are taken from ref (62). DFT labels at the B3LYP level of theory are taken from ref (60) for *n*-alkanes and *n*-alkenes. Test molecule geometries are generated with the RDKit library^55^ for *n*-alkanes, and taken from the QM7b data set for *n*-alkenes.

The QM7b data set^60^ consists of polarizability tensors from 7211 organic molecules, here we use the polarizabilities calculated with the B3LYP functional^48^ and the d-Aug-cc-pVDZ basis set.^61^ This data set was used to train the CRK and the polarizability tensor **α** based on eq 9. In this setup, any planar molecule would have a zero diagonal component in the polarizability tensor. Therefore, we measure the extent of molecules by their thickness, which we define as the variance σ_*z*_^2^ in position *R*_*z*_. By this definition, a molecule with bonds only in the xy plane has σ_*z*_^2^ = 0. The QM7b data set is visualized in terms of molecular thickness in the upper panels of Figure 4a,b. The lower panels of these plots show the prediction accuracy as a function of molecular thickness for PiNet-χ and PiNet2-χ, respectively. PiNet2-χ outperforms PiNet-χ in all models except the EEM-type (also see Table S8, where PiNet2-χ does better than PiNet-χ when molecules within a cutoff thickness were excluded).

Besides the prediction accuracy, the scaling behavior of polarizability is equally important. A well-known issue that EEM-type models suffer from is the superlinear scaling of the polarizability with molecular size.^63^ To investigate the scaling behavior of our models, we predicted the polarizability of *n*-alkane and *n*-alkene chains. The predicted isotropic polarizability α_iso_ is plotted versus the number of carbon atoms *n*_C_ of *n*-alkanes in Figure 4c,d and for *n*-alkenes in Figure 4e,f. In the PiNet-χ prediction for the EEM-type model, we see a superlinear deviation from the expected linear behavior for *n*-alkanes when the molecular size is outside of the training set. However, for PiNet2-χ, EEM no longer overestimates the polarizability of *n*-alkanes, and the scaling behavior is linear even for structures well outside of the training set. Therefore, what we found here is that the EEM-type model powered by PiNet2-χ is now able to capture the correct physical scaling in polarizability for both conjugated and nonconjugated systems.

### 4.5 Liquid Water and Electrolyte Solution Data Sets: H_2_O(l)-revPBE0-D3 and NaCl(sol)-SCAN

To further test the performance of PiNet2-P3, we have also applied both PiNet and PiNet2-P3 to generate MLPs for liquid systems, starting with the liquid water data set. This data set that we call “H_2_O(l)-revPBE0-D3”, published by Cheng et al.,^64^ comprises 1593 liquid water configurations involving 64 molecules with the energies and forces computed by DFT at the revPBE0-D3 level^65−67^ and presents an excellent example to test MLPs in the interpolation regime.^68^ To prevent overfitting and improve training efficiency, smaller layers in both PiNet and PiNet2-P3 were employed (with 16 nodes per layer instead of 64) and details about the choices of hyperparameters can be found in the Supporting Information. The invariant PiNet reaches an RMSE of 9.57 meV/H_2_O for energy and 146.74 meV/Å for force, which is slightly worse than the original results obtained with the Behler–Parrinello neural network (BPNN),^69^ i.e., RMSEs for energy and force are 7.0 meV/H_2_O and 120 meV/Å. In comparison, the corresponding RMSEs dropped to 5.97 meV/H_2_O for energy and 90.71 meV/Å for force in the case of PiNet2-P3.

**Figure 5:**
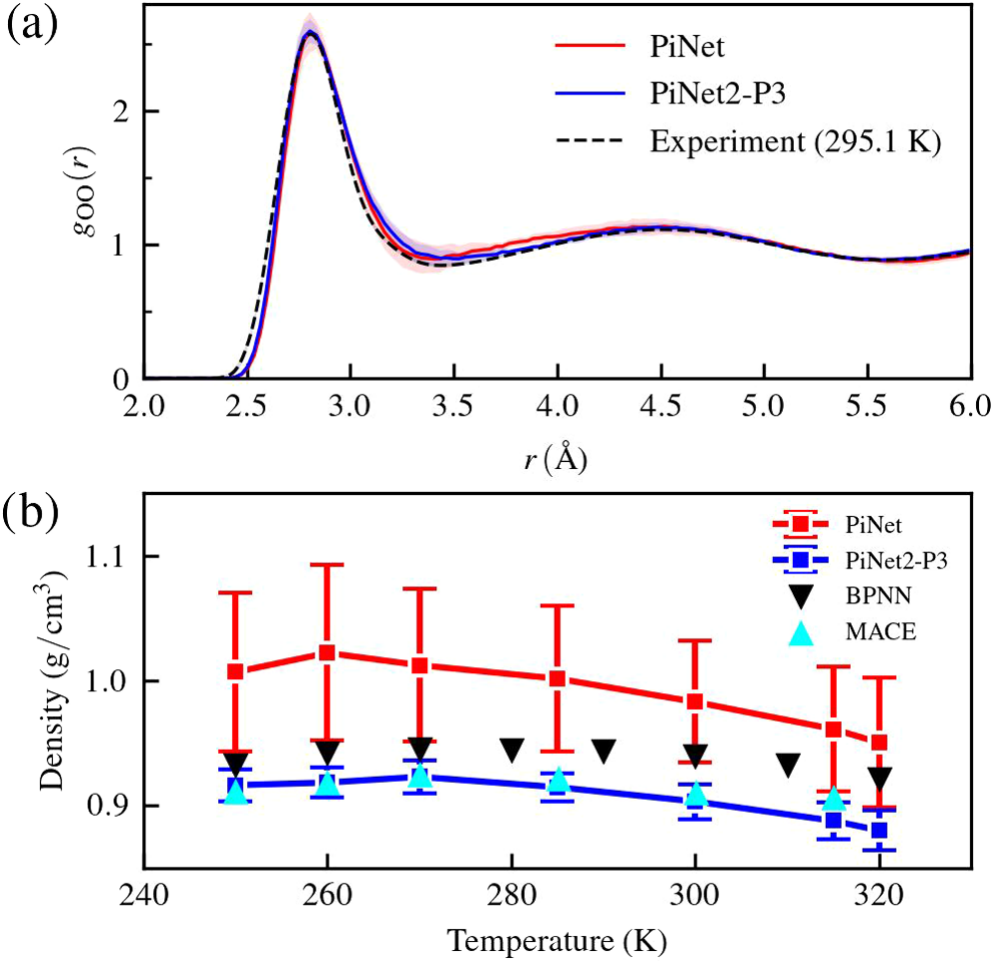
Performance of MLPs from PiNet and PiNet2-P3 trained on the “H_2_O(l)-revPBE0-D3” data set. (a) O–O radial distribution functions *g*_OO_(*r*) of liquid water at 300 K and 0.996 g/cm^3^ in the *NVT* ensemble together with the experimental ref (70) at 295.1 K. The standard deviations obtained from 10 independent models are shown in transparent colors. (b) Temperature dependence of equilibrium densities at 1.0 bar in the *NPT* ensemble. The standard deviations were obtained from 10 independent models. Results from BPNN^64^ and MACE^71^ are also shown.

When it comes to the actual performance beyond error metrics, it is found that the calculated radial distribution functions (RDFs) obtained from PiNet and PiNet2-P3 are quite similar in the constant number of particles, volume, and temperature (*NVT*) ensemble. They also agree well with the experimental results (Figure 5a). However, things are quite different for the temperature dependence of the density in the constant number of particle, pressure, and temperature (*NPT*) ensembles as shown in Figure 5b. The equilibrium densities were very much overestimated with PiNet while densities predicted from PiNet2-P3 agree surprisingly well with those reported by MACE.^71^ Therefore, it becomes clear that equivariant features play a critical role in MLPs for describing the structure and the thermodynamics of liquids, and PiNet2-P3 appears to be very capable of describing this type of systems.

Real electrolyte solutions contain ions; therefore, we have also tested the performance of PiNet2-P3 using the data set that we call “NaCl(sol)-SCAN”. This data set^72^ contains 26926 structures of NaCl aqueous solutions at a variety of concentrations, while the energy and force labels were obtained through DFT calculations with the SCAN functional.^73^ RMSEs are 1.19 meV/atom for energy and 70 meV/Å for force for PiNet2-P3, which are much smaller than the corresponding values obtained for PiNet, i.e., 2.17 meV/atom for energy and 126 meV/Å. These numbers can be compared to the RMSEs of 0.3 meV/atom for energy and 72.4 meV/Å from DeePMD in ref (72) where the original data set comes from.

**Figure 6:**
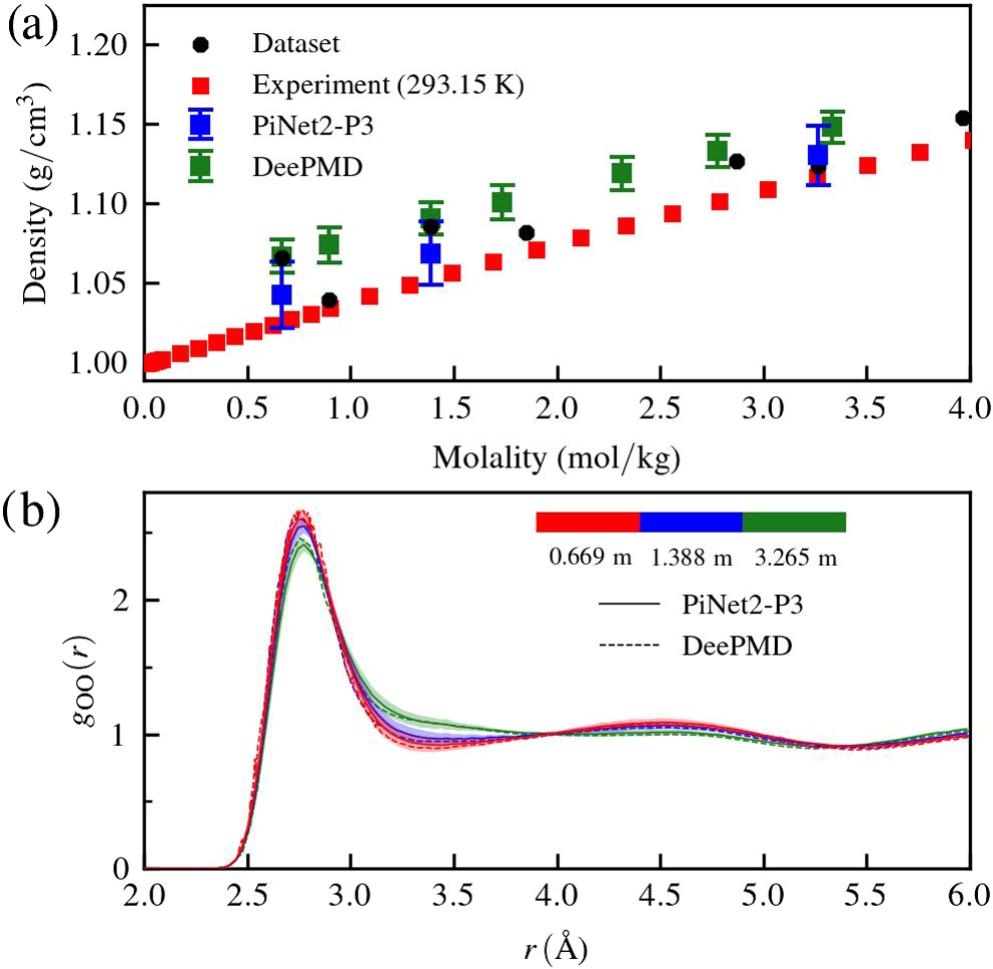
Performance of MLPs from PiNet and PiNet2-P3 trained on the “NaCl(sol)-SCAN” data set. (a) Predicted equilibrium densities at three salt concentrations, 333 K and 1.0 bar in the *NPT* ensemble. The standard deviations were obtained from 7 independent models. The average densities of the data set, the results from DeePMD,^72^ and the experimental densities at 293.15 K were also shown. (b) O–O radial distribution functions *g*_OO_(*r*) of NaCl solutions at 333 K and 1.0 bar in the *NPT* ensemble. The standard deviations obtained from 7 independent models are shown in transparent colors. The results of DeePMD^72^ are shown as reference.

The actual performance of MLPs obtained from PiNet2-P3 is shown in Figure 6. As compared to DeePMD, PiNet2-P3 reproduces not only the correct concentration-dependent densities (Figure 6a) but also the modulation of the water structure in concentrated electrolyte solutions (Figure 6b). To go the extra mile, we have applied this set of MLPs trained with PiNet2-P3 to calculate the ionic conductivity at 330 K with the Bussi–Donadio–Parrinello thermostat.^74^ The predicted ionic conductivities of the NaCl solutions (obtained from the diffusive regime of the corresponding polarization mean squared displacement) show a very encouraging agreement with experimental reference in terms of both the concentration-dependence and the absolute value (Figure S3 in the Supporting Information), although it is much more challenging to converge this quantity at a higher concentration.

### 4.6 Application of PiNNAcLe to Simulate Protic Ionic Liquids

To demonstrate the capability of PiNet2-P3 and PiNNAcLe for realistic applications, we focus here on the structural and transport properties of the protic ionic liquid (PIL) [C_1_IM][HOAc], where ion pairs form upon the proton transfer (PT) reaction between the Brønsted acid/base pair.^75,76^

Intriguing experimental observations were made for this system, including the divergent estimation of the degree of PT reaction,^76,77^ and the unexpected high conductivity, leads to discussion of the mechanism of proton conduction, where a Grotthus-type specific proton conduction is conjectured.^78,79^

**Figure 7:**
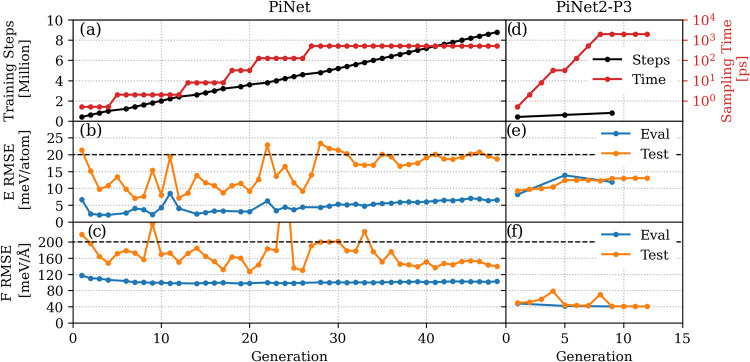
Performance of PiNet and PiNet2-P3 models on the AcLe task for the protic ionic liquid system in the case of the SCAN functional. (a, d) Convergence of energy and force errors for each generation, and time scale of the sampled trajectory; (b, e) energy errors on the validation data during the training step and the test data during the sampling step; (c, f) force errors on the validation data during the training step and the test data during the sampling step. Dashed lines indicate energy and force tolerances set in this AcLe task.

For this case study, we compare the performance of PiNet and PiNet2-P3 and combine them with different reference DFT calculations. Specifically, we have used the revPBE functional^65^ with Grimme’s dispersion correction^80^ and the SCAN functional.^73^ A detailed description of the reference electronic structure calculations can be found in the Supporting Information. It is found that RMSEs for energy and force from PiNet2-P3/SCAN are about 11.9 and 40.7 meV/Å, respectively, as compared to those of 7.2 and 102.8 meV/Å of PiNet/SCAN. A significant reduction in the force errors is also seen in the case of the revPBE-D3 functional (see Table S14 in the Supporting Information). For the adaptive learn-on-the-fly (AcLe) task, a more critical performance metric is how well the MLPs perform on the newly sampled MD trajectories, i.e., the test error. Figure 7 shows that the test error of MLPs from PiNet2-P3 converges much faster as compared to those from PiNet. In other words, it requires much less generations in the AcLe algorithm to obtain long and stable MD trajectories with PiNet2-P3. This suggests that the inclusion of P3 features helps greatly with the generalization of MLPs.

**Figure 8:**
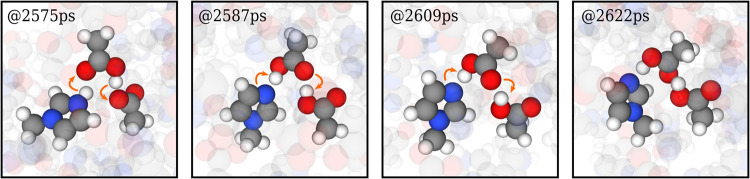
MD simulation snapshots from MLPs generated with PiNet2-P3/SCAN showcasing ion transport through concerted proton transfer events in the protic ionic liquid [C_1_IM][HOAc]. The arrows indicate the proton transfer directions.

Subsequently, we performed long MD simulations of 5 ns (in order to reach the diffusive regime) with the last generations of MLPs to compute transport coefficients. Following ref (81), the conductivity is computed using the Green–Kubo formula by assigning fixed integer charges to the charged species (mobile protons and acetate groups). The results are shown in Table 4. We found that PiNet2-P3/SCAN better reproduces the self-diffusion coefficient, the Green–Kubo conductivity, and the Nernst–Einstein deviation parameter Δ reported by experiments, as compared to PiNet2-P3/revPBE-D3. The fractions of neural acetic acid were identified as 0.87(2) and 0.78(1) from PiNet2-P3/revPBE-D3 and PiNet2-P3/SCAN, respectively. Taking these fractions and constraining active hydrogen atoms to not undergo PT reactions, the resulting Green–Kubo conductivities were as low as one-third of those from the same systems that allows PT reactions. This provides supporting evidence that a long distance and concerted PT (see Figure 8) accounting for the fast ion transport in the protic ionic liquid [C_1_IM][HOAc] is indeed possible, although more detailed studies are needed to fully confirm this point, which is beyond the scope of the showcase demonstrated here.

**Table 4:** Self-Diffusion Coefficients *D*^s^ (10^–2^ Å^2^ ps^–1^) of Active Protons and Acetate Groups, the Green–Kubo Conductivity σ_G–K_ (mS cm^–1^), and the Nernst–Einstein Deviation Δ = 1 – σ_G–K_/σ_N–E_ Obtained from MD Simulations at 340 K Using MLPs Generated with PiNet2-P3 (Abbreviated as “P3” in the Table) and PiNNAcLe^a^

model	*D*_C_1_Im_^s^	*D*_OAc_^s^	σ_G–K_	Δ
P3/revPBE-D3	1.06(7)	0.97(9)	6(1)	0.89
P3/revPBE-D3^b^	0.9(1)	0.7(2)	4(1)	0.91
P3/SCAN	3.7(1)	3.1(1)	14(6)	0.92
P3/SCAN^b^	3.4(3)	3.0(3)	5(3)	0.97
exp.	5.84	5.09	11.9	0.96

a Experimental density^82^ was used in these simulations.

b Harmonic constraints have been applied to any covalent bond containing hydrogen. Active proton and PT reactions were thereby prohibited in the corresponding simulations. Experimental results at 333 K come from ref (78).

### 4.7 Application of PiNNwall to Simulate Polarized Graphene Oxide Electrode

**Figure 9:**
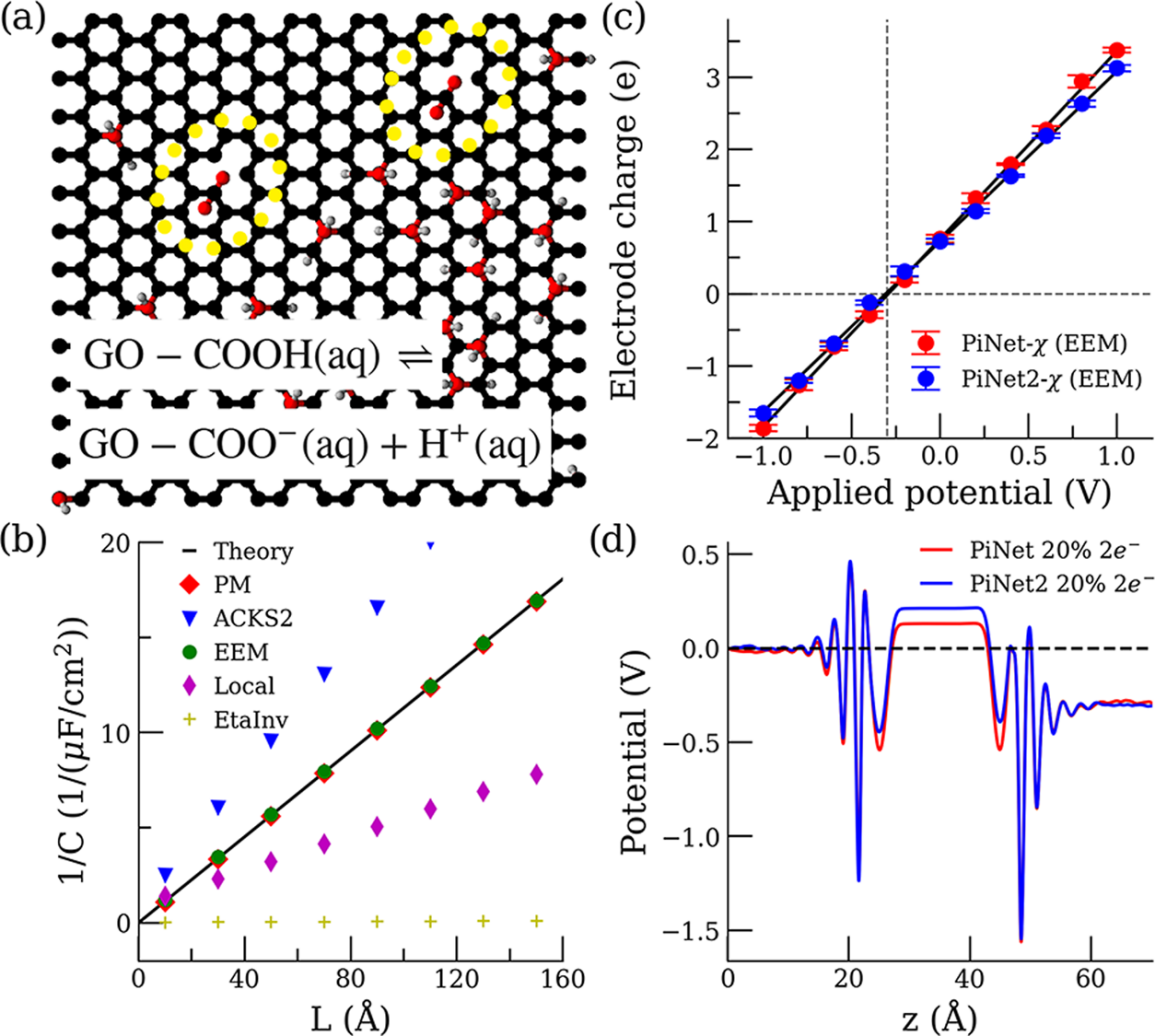
Molecular simulations of electrified graphene oxide/electrolyte interfaces. (a) Snapshot of the carboxyl-terminated electrode surface with a 10% surface coverage of OH. Electrolyte solution is not shown for the sake of clarity. The acid–base reaction considered for carboxyl groups is indicated. (b) Validation test of the inverse capacitance 1/*C* of an empty capacitor (graphite electrode) as a function of the vacuum slab size *L*. (c) Electrode charge as a function of applied potential. *V*_PZEC_ is identified when the electrode charge becomes zero. (d) Electrostatic potentials of the graphene oxide electrode with a proton charge of 2e^–^ and 20% surface coverage of the hydroxyl interfaced at the *V*_PZEC_.

To demonstrate the capability of PiNet2-χ, PiNet2-dipole, and PiNNwall for realistic applications, we showcase here the molecular simulations of electrified graphene oxide/electrolyte interfaces. In graphene oxide, both surface carboxylic and hydroxyl groups can undergo protonation/deprotonation depending on the solution pH. It has been reported that the p*K*_a_ is about 6.6 for the carboxylic group and 9.8 for the hydroxyl group in graphene oxide.^83^ Therefore, graphene oxide is negatively charged at neutral pH through the acid–base reaction shown in Figure 9a, and this property contributes to the stability of graphene oxide membranes in aqueous solutions.^84^

Before turning on the MD engine for simulating the electrode–electrolyte system, we checked the predictions from PiNet2-χ regarding the response charge for the case where the analytical solution is available. This is the case of a graphite electrode in vacuum. We used this system to compute the corresponding capacitance by varying the thickness of the vacuum slab. When the electrode behaves like a perfect metal with a dielectric constant of infinity, the total capacitance of the whole system (electrode slab + vacuum) will only depend on the thickness of the vacuum. The results of the different models implemented in PiNet2-χ are shown in Figure 9b. It is clear that the EEM-type model reproduces the expected theoretical behavior of a perfect metallic electrode, which is not the case for the ACKS2-type, the Local-type, and the EtaInv-type models. Therefore, we have selected the EEM-type model as the charge response kernel for the application here.

To locate the point of zero electrode charge (PZEC), we applied a series of external potentials to the graphene oxide/electrolyte system. Results of this “titration” procedure are shown in Figure 9c. Comparing PiNet and PiNet2, the PZECs are almost identical and are about −0.30 V. The corresponding electrostatic potential profile of the system is shown in Figure 9d. Despite having almost the same PZECs, the contributions from two sides of electrode to the overall potential drop are not the same between PiNet and PiNet2. This raises the question of whether this difference led by PiNet2 contributes to the capacitance asymmetry which are often found in the aqueous electrolyte solutions and suggested to correlate with water adsorption energy.^85^

**Figure 10:**
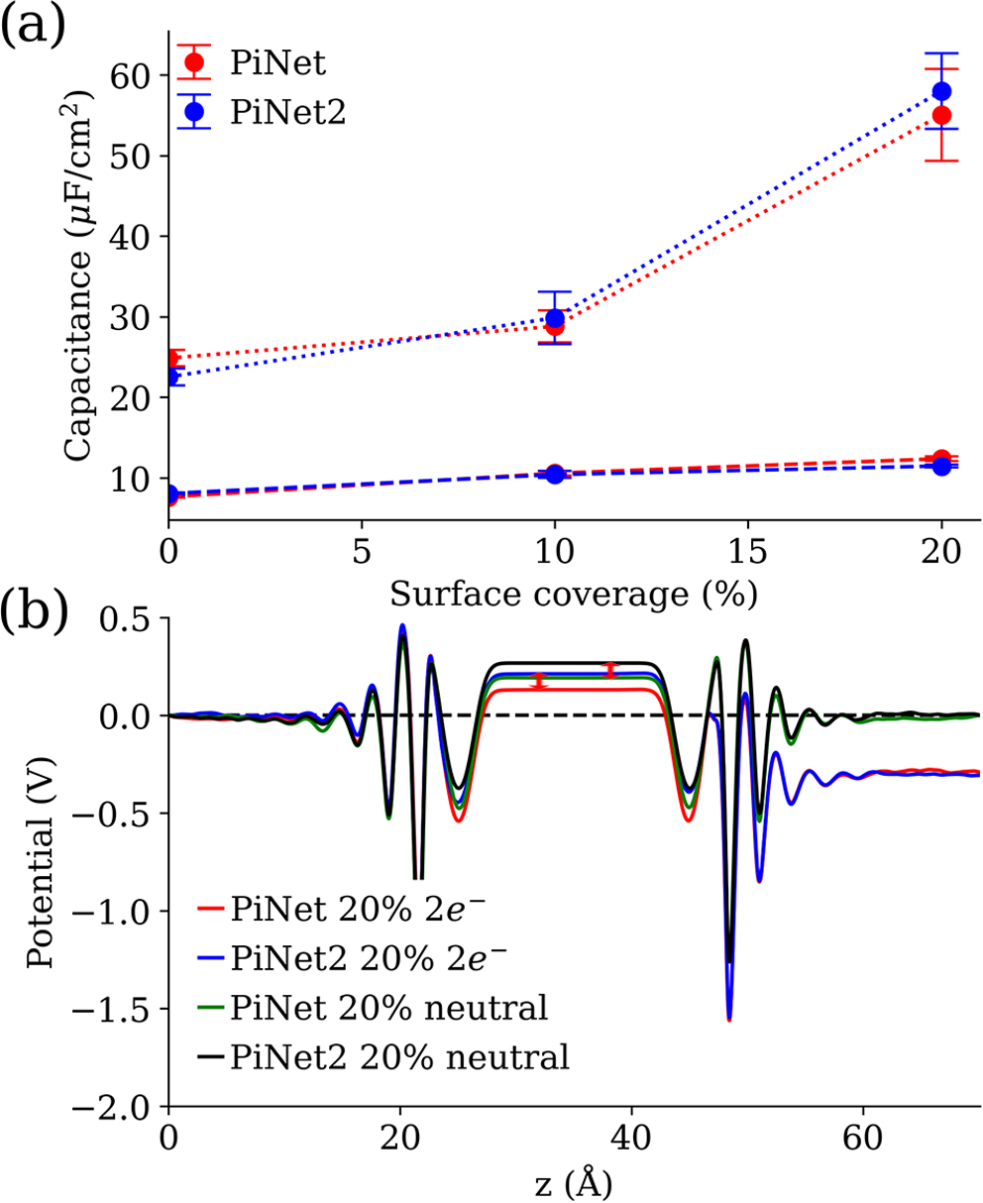
Analysis of the Helmholtz capacitance and the electrostatic potential profiles of graphene oxide/electrolyte interfaces. (a) Helmholtz capacitance for the positive (dashed line) and negative electrodes (dotted line) as a function of the surface coverage of OH. (b) Comparison of the electrostatic potential profiles of the graphene oxide electrode with 20% surface coverage of hydroxyl groups at PZEC from PiNet and PiNet2 with and without a proton charge of 2e^–^ from the deprotonated carboxyl groups.

In Figure 10, the capacitance as it changes with hydroxyl surface coverage for the PiNet and PiNet2 models and the electrostatic potential profiles for the 20% surface coverage case are shown. Despite the fact that the potential profiles in Figure 9d seem to shift by about 80 mV when switching from PiNet to PiNet2, the resulting capacitance remains almost the same. It should be pointed out that the Helmholtz capacitance reported here appear to be half of that reported previously.^26^ This is due to a missing factor of 2 in the conversion instead of any physical difference in the simulation systems. Nevertheless, the main conclusion remains the same that the proton charge can increase the Helmholtz capacitance at PZEC significantly, which becomes comparable in magnitude with respect to those found in the metal-oxide-based systems for protonic double layer.^85,86^

Comparing the electrostatic potential profiles of the charged system of PiNet and PiNet2 with those of the corresponding neutral system, it is found that there is a constant shift in these two cases as a result of the differing base charges. Therefore, this shift simply cancels out when computing the capacitance, because only the potential difference between the neutral and the charged surfaces matters. This highlights the fact that the Helmholtz capacitance is a response property.

## 5 Conclusions and Outlook

In this work, we successfully upgraded our atomistic ML suite PiNN to include equivariant features in the PiNet2 architecture. Through careful tests and benchmarks using publicly available data sets of small molecules, crystalline materials, and liquid systems, it is found that PiNet2-P3 shows a universal and significant improvement with moderate computational overhead as compared to PiNet. This does not only apply to energy and force predictions but also to dipole, charge, and polarizability predictions, as demonstrated by the PiNet2-dipole and PiNet2-χ modules. New versions of the PiNN code are freely accessible and kept updated at https://github.com/Teoroo-CMC/PiNN with documentation and tutorials.

To showcase the capabilities of PiNN for modeling electrochemical systems, we have applied PiNet2-P3 with the adaptive learn-on-the-fly plug-in PiNNAcLe to generate MLPs for protic ionic liquid [C_1_IM][HOAc] and PiNet2-χ/PiNet2-dipole with the MetalWalls plug-in PiNNwall to carry out molecular simulations of electrified graphene oxide/electrolyte interfaces. These allow us to study the role of long-distance PT in ionic conductivity with reactive MD simulations and to investigate the role of proton charge in the Helmholtz capacitance of aqueous supercapacitors under external bias.

Since the introduction of the idea to couple finite-field methods and atomistic ML,^87,88^ a number of implementations have been developed and applied to simulate insulating condensed-phase systems (e.g., liquid water) with field-dependent MLPs.^89−91^ In the meantime, the integration of atomistic ML and molecular simulation for modeling metallic electrodes has also been made possible, as exemplified by the development of PiNNwall and other upcoming and interesting “QM/MM”-type implementations with ML ingredients.^92−94^ Very recently, finite-field DFTMD simulations have been demonstrated for modeling polarized Au(100) and Au(111) electrodes immersed in electrolyte solution.^95^ This long-awaited endeavor opens the door for joining these two streams in the near future for a full integration and ML description of reactive and polarizable electrochemical interfaces. With the equivariant neural network suite PiNN introduced here, we are ready for this challenge and anticipating many interesting applications to realistic electrochemical systems coming out soon.

## Supporting Information Available

Computational setups and hyperparameter tables for all case studies, the benchmark plot of execution time from PiNet and PiNet2, error metrics in RMSE for both QM9 and rMD17 data sets, comparison of PiNet-dipole and PiNet2-dipole families for the QM9 data set, predicted ionic conductivities of NaCl solutions from MLP-based MD simulations with PiNet2-P3/SCAN, comparison of transport coefficients from MLP-based MD simulations with PiNet and PiNet2-P3 for protic ionic liquids, and base charges predicted from PiNet2-dipole for graphene oxide electrode with different terminations (PDF)
