# Supplementary material for: PiNN: Equivariant Neural Network Suite for Modeling Electrochemical Systems
Source: J Chem Theory Comput. 2025 Jan 30;21(3):1382–95. doi: 10.1021/acs.jctc.4c01570 (PMC11823406; doi:10.1021/acs.jctc.4c01570)
Supplement: Supplementary file 1 — ct4c01570_si_001.pdf [file ct4c01570_si_001.pdf]

Supporting Information: PiNN: equivariant neural network suite for modelling electrochemical systems

Jichen Li,[†,¶] Lisanne Knijff,[†,¶] Zhan-Yun Zhang,[†,‡] Linnéa Andersson,[†] and Chao Zhang[*,†,‡]

†*Department of Chemistry-Ångström Laboratory, Uppsala University, Lägerhyddsvägen 1, P. O. Box 538, 75121 Uppsala, Sweden*

‡*Wallenberg Initiative Materials Science for Sustainability, Uppsala University, 75121 Uppsala, Sweden*

¶*Contributed equally to this work*

E-mail: chao.zhang@kemi.uu.se

Hyperparameters and execution time for crystalline materials datasets

The hyperparameters of both Materials Project (MP) and Perovskites benchmarks are detailed in Table S1. Hyperbolic tangent activation functions were applied to all invariant layers except the output layer, where a linear activation function was used. The batch size for MP-crystals-2018.6.1 was set identical to that in the original PiNN paper,[S1] while the batch size for MPF.2021.2.8 matched that of M3GNet. An 80:20 dataset split was implemented for case studies, meaning 80% of each dataset's structures were randomly selected for training, and the remaining 20% were used for validation. We employed the Adam optimizer in TensorFlow for gradient descent updates, setting the learning rate to 0.0001 and decaying it by a factor of 0.994 every 100,000 steps. To prevent exploding gradients, a gradient norm clipping strategy was adopted. Training was terminated after 3 million gradient descent steps when the evaluation curves had converged.

Table S1: Hyperparameters used to train the PiNet, PiNet2-P3 and PiNet2-P5 models for the Material Project datasets. Here R_c is the interaction cut-off, and n_{basis} is the number of basis functions for each (i,j) tuple.

Layer	Architecture	Parameter	Value
PI	[64, 64] $\times$ 10	R_c	6 Å
II	[64, 64, 64, 64]	GC blocks	4
PP	[64, 64, 64, 64]	n_{basis}	10
Output	[64, 64,1]		

To assess the associated computational overhead introduced by equivariant features, we evaluated the training and inference speeds on the MPF.2021.2.8 dataset using a single NVIDIA A100 GPU. Compared to the enhanced performance, the additional computational overhead remains within an acceptable range.

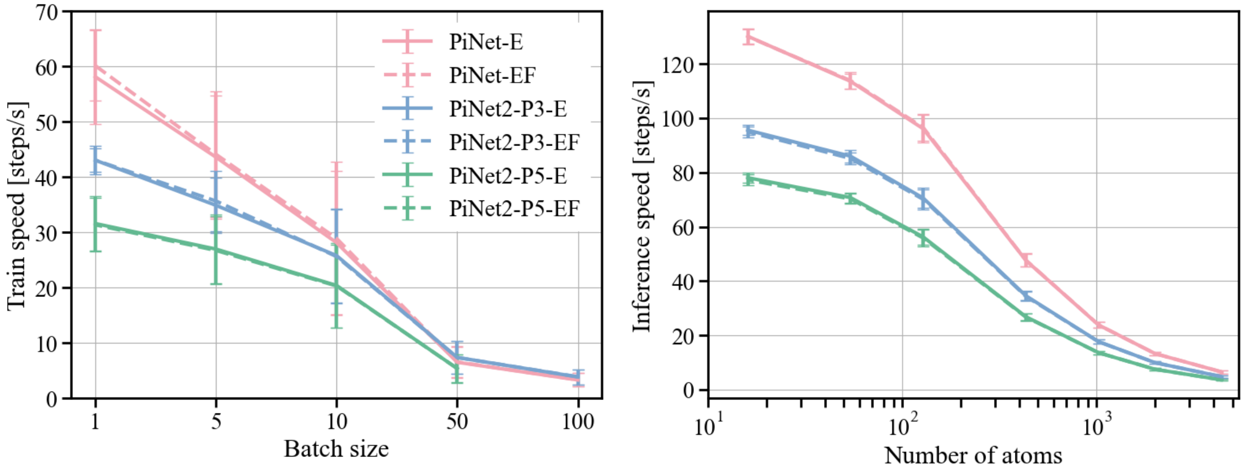

Figure S1: Training and inference benchmarks on the MPF.2021.2.8 dataset using a single A100 GPU card.

Hyperparameters and error metrics in RMSE for the QM9 and the MD17 datasets

For batch size, we have used 100 for the QM9 dataset, following the original PiNN paper,[S1] and 10 for the MD17, as recommended by the PaiNN paper.[S2] For dataset splits, an 80:20 ratio was applied to the QM9 dataset, with 80% of the structures randomly allocated for training and 20% for validation. For MD17, 950 configurations were designated for training and 50 for validation, consistent with the approach taken in the PaiNN paper. PiNet2-P3 achieves an RMSE of 17 ± 1 meV in predicting internal energy at 0K, compared to 25 ± 2 meV RMSE of the PiNet result for the same data splitting and hyperparameters.

The hyperparameters for the QM9 and MD17 datasets are presented in Table S2. All invariant layers used hyperbolic tangent activation functions, except for the output layer, which applied a linear activation. The Adam optimizer in TensorFlow was employed for gradient descent, with an initial learning rate of 0.0001 that decayed by a factor of 0.994 every 100,000 steps. A gradient norm clipping was applied to mitigate the risk of exploding gradients. Training terminated after 1 million steps when evaluation curves indicated convergence.

Table S2: Hyperparameters used to train the PiNet and PiNet2-P3 models for QM9 and MD17 datasets. Here R_c is the interaction cut-off, and n_{basis} is the number of basis functions for each (i,j) tuple.

Layer	Architecture	Parameter	Value
PI	[64] × 10	R_c	4.5 Å
II	[64, 64, 64, 64]	GC blocks	5
PP	[64, 64, 64, 64]	n_{basis}	10
Output	[64, 1]		

Table S3: Root-mean-square deviation errors (RMSEs) for energy and force from invariant PiNet and equivariant PiNet2-P3 trained on the rMD17 datasets. We have used 950 training samples and 50 validation samples in model training. For each molecule and network architecture, three independent models were trained with random dataset splits but using the same ratio.

energy (meV)	trained on forces only		trained on energy & forces	
force (meV/Å)	PiNet	PiNet2-P3	PiNet	PiNet2-P3
Aspirin	-	-	20(4)	6.9(1)
	68(2)	19(3)	77(4)	29(6)
Ethanol	-	-	1.8(4)	1.6(5)
	18(6)	9.9(1)	16(1)	12(4)
Malonaldehyde	-	-	4.8(5)	1.8(6)
	28(9)	11(1)	22(2)	12(1)
Naphthalene	-	-	10.2(9)	1.6(7)
	46(3)	3.1(4)	48(3)	5.0(2)
Salicylic acid	-	-	11.1(8)	3(1)
	69(12)	12(2)	66(3)	15(4)
Toluene	-	-	9(2)	1.7(6)
	34(2)	4(2)	40(3)	6(1)
Uracil	-	-	13(8)	1.5(3)
	48(18)	6(1)	61(22)	9(1)

Hyperparameters and dipole predictions for the QM9 dataset

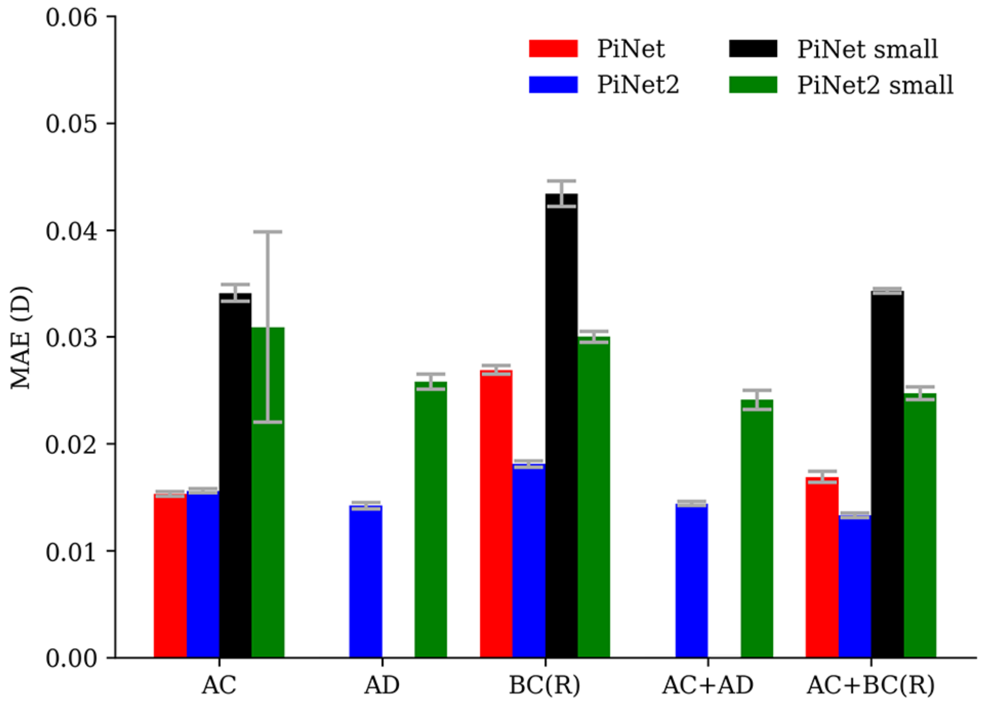

Figure S2: Plot showing the mean average error (MAE) of the PiNet-dipole and PiNet2-dipole models of different variants when trained on the QM9 dataset. This is done for networks of a small size and a larger size for which the hyperparameters can be found in Table S5 and S4 respectively. Results are shown for the atomic charge (AC) model, atomic dipole (AD) model, bond charge model with regularization applied (BC(R)), and two models which combine these components, AC+AD and AC+BC(R).

Table S4: Hyperparameters used to train the PiNet-dipole and PiNet2-dipole models for the QM9 dataset. Here R_c is the interaction cut-off, and n_{basis} is the number of basis functions for each (i,j) tuple.

Layer	Architecture	Parameter	Value
PI	[64] × 10	R_c	4.5 Å
II	[64,64,64,64]	GC blocks	5
PP	[64,64,64,64]	n_{basis}	10
Output	[64,1]		

Table S5: Hyperparameters used to train the PiNet-dipole and PiNet2-dipole models for the QM9 dataset for the small network size. Here R_c is the interaction cut-off, and n_{basis} is the number of basis functions for each (i,j) tuple.

Layer	Architecture	Parameter	Value
PI	[16, 16] × 10	R_c	4.5 Å
II	[16, 16]	GC blocks	5
PP	[16, 16,]	n_{basis}	10
Output	[16, 16, ,1]		

In both cases an 80:20 dataset split was used for the training and testing respectively. A batch size of 100 samples was used for the training. The Adam optimizer was used with a learning rate to 0.0001 and decaying it by a factor of 0.994 every 100,000 steps. To prevent exploding gradients, a gradient norm clipping strategy was adopted. Training was terminated after 3 million gradient descent steps.

Hyperparameters for training the supercell dipole of LIQWAT dataset and the computation of IR spectra

Table S6: Hyperparameters used to train the PiNet-dipole and PiNet2-dipole models for the LIQWAT dataset. Here R_c is the interaction cut-off, and n_{basis} is the number of basis functions for each (i,j) tuple.

Layer	Architecture	Parameter	Value
PI	[64] × 10	R_c	4.5 Å
II	[64,64,64,64]	GC blocks	5
PP	[64,64,64,64]	n_{basis}	10
Output	[64,1]		

In both cases a 80:20 dataset split was used for the training and testing respectively. A batch size of 64 samples was used for the training. The Adam optimizer was used with a learning rate to 0.0001 and decaying it by a factor of 0.994 every 100,000 steps. To prevent exploding gradients, a gradient norm clipping strategy was adopted. Training was terminated after 5 million gradient descent steps, or early if overfitting started to occur.

The IR spectra are calculated using the total dipole predicted by the PiNet2-dipole models with the TRAVIS code[S3,S4] according to Eq. 1.

$$I(\omega) \propto \omega^2 \int_{-\infty}^{\infty} dt e^{-i\omega t} \langle \mathbf{M}(0)\mathbf{M}(t) \rangle \tag{1}$$

where $\mathbf{M}$ is the supercell dipole.

The spectra are computed using a depth of the autocorrelation function of 1024. To enhance the spectra, a Hann window function and zero-padding were applied to the autocorrelation function. The MD trajectory of liquid water was sampled with PiNet2-P3/revPBE0-D3 at 300 K and experimental density. A timestep of 0.5 fs was used and the trajectory was saved every 2 fs.

Hyperparameters for training the polarizability of QM7b

The PiNet-χ and PiNet2-χ models for the polarizability tensor were trained on 80 % of the entire QM7b dataset. The presented results in table S9 and Figure 4 were averaged over 10 training instances for each type of model. The training was performed using the Adam optimizer[S5] in TensorFlow[S6] for gradient descent updates, with the initial learning rate 3×10^{-4} and a decay factor of 0.994 every 10^4 steps. The models were trained for 5×10^5 steps with a mini-batch size of 30. To prevent exploding gradients, a gradient norm clipping strategy was adopted.[S7] The network hyperparameters are presented in Tables S7 and S8.

Table S7: Hyperparameters used to train PiNet-χ models for polarizability tensor prediction on the QM7b dataset. Here R_c is the interaction cut-off, n_{basis} is the number of basis functions for each (i,j) tuple and η is the basis function Gaussian width.

Layer	Architecture	Parameter	Value
PI	[32] × 20	R_c	4.5 Å
II	[32,32,32,32]	GC blocks	5
PP	[32,32,32,32]	n_{basis}	20
PI_{out}	[32] × 20	η	3 Å^{-2}
PP_{out}	[32,1]		

Table S8: Hyperparameters used to train PiNet2-χ models for polarizability tensor prediction on the QM7b dataset. Here R_c is the interaction cut-off, n_{basis} is the number of basis functions for each (i,j) tuple and η is the basis function Gaussian width.

Layer	Architecture	Parameter	Value
PI	[32] × 20	R_c	4.5 Å
II	[32,32,32,32]	GC blocks	5
PP	[32,32,32,32]	n_{basis}	20
Output	[32,1]	η	3 Å^{-2}

Table S9: Errors metrics for PiNet-χ and PiNet2-χ models for polarizability tensor α. Presented here is the prediction error for the QM7b dataset excluding the training data and molecules with a thickness σ_z^2 smaller than 4 Å^2. Errors are presented as RMSE per atom (a.u.) for the full tensor as well as its isotropic and anisotropic parts. The results from PiNet are taken from ref S8 .

Model	RMSE	RMSE_{iso}	$\text{RMSE}_{\text{aniso}}$
EEM (PiNet2-P3)	0.28(4)	0.13(2)	0.24(4)
ACKS2 (PiNet2-P3)	0.083(5)	0.048(4)	0.067(4)
EtaInv (PiNet2-P3)	0.11(1)	0.06(2)	0.082(4)
Local (PiNet2-P3)	0.11(2)	0.08(3)	0.083(7)
EEM (PiNet)	0.4 (1)	0.3 (1)	0.33 (8)
ACKS2 (PiNet)	0.11 (1)	0.07 (1)	0.086 (6)
EtaInv (PiNet)	0.12 (1)	0.07 (2)	0.102 (6)
Local (PiNet)	0.15 (2)	0.10 (3)	0.12 (1)

Hyperparameters for training potential energy surfaces of liquid water and electrolyte solution

Table S10: Hyperparameters of PiNet and PiNet2-P3 models on the H2O(l)-revPBE0-D3 and NaCl(sol)-SCAN datasets.

Layer	Architecture	Parameter	Value
PI	[16] $\times$ 10	R_c	6.0 Å
II	[16,16,16,16]	GC blocks	5
PP	[16,16,16,16]	n_{basis}	10
Output	[16,1]		

Hyperparameters used to train MLPs on the H2O(l)-revPBE0-D3 and NaCl(sol)-SCAN datasets are listed in Table S10. A total of 5 million gradient descent steps were performed using the Adam optimizer with a batch size of 1. The learning rate was reduced from 0.00005 every 100,000 steps at a decay rate of 0.994. In addition, weights of energy and force components in the loss function were set as 10.0 and 100.0, respectively. Finally, ten PiNet/PiNet2-P3 models were separately constructed on the H2O(l)-revPBE0-D3 dataset using different random data splitting but the same ratio, while seven PiNet/PiNet2-P3 models were built on the NaCl(sol)-SCAN dataset.

For radial distribution functions (RDFs) and equilibrium densities, MLP-based MD sim-

ulations were performed with the Berendsen thermostat and barostat[S9] implemented in the ASE package,[S10] and the time step was set as 0.5 fs. The number of independent trajectories were generated for each system matches the respective number of models (i.e., one trajectory corresponds to one PiNet/PiNet2-P3 model). On the liquid water system, the O-O RDFs were calculated from 200 ps long NVT trajectories at 300 K, while the density isobars were obtained from 200 ps long NPT trajectories at 1.0 bar. On the NaCl aqueous solution systems, both densities and O-O RDFs were obtained from 2 ns long NPT trajectories at 333 K and 1.0 bar. The initial stoichiometries of boxes used in this case are summarized in Table S11.

For ionic conductivities, the Berendsen thermostat was replaced by the Bussi-Donadio-Parrinello thermostat[S11] which has been popularly used to simulate dynamic properties.[S12] Much longer NVT trajectories (i.e., 5 ns) at 330K were collected to guarantee the convergence of ion diffusions. The final ionic conductivities were calculated by the Green-Kubo equation

$$\sigma = \lim_{t \to \infty} \frac{\beta V}{6t} \left\langle [\mathbf{P}(t) - \mathbf{P}(0)]^2 \right\rangle \tag{2}$$

where $\beta = 1/k_b T$ is the inverse temperature, V is the system volume, $\mathbf{P}$ is the itinerant polarization of system.[S12] The stoichiometries of boxes used in this case are summarized in Table S12.

Table S11: The initial stoichiometries of boxes used in MLP-based MD simulations for radial distribution functions and densities.

N_{H_2O}	N_{NaCl}	Cubic cell length (Å)	Molality (mol/kg)	Density (g/cm^3)
64	0	12.43	0	0.996
83	1	13.54	0.669	1.039
80	2	13.53	1.388	1.046
68	4	13.03	3.265	1.096

Table S12: The stoichiometries of boxes used in MLP-based MD simulations for ionic conductivities.

N_{H_2O}	N_{NaCl}	Cubic cell length (Å)	Molality (mol/kg)	Density (g/cm^3)
496	8	24.72	0.896	1.033
480	16	24.60	1.852	1.069
448	32	24.39	3.968	1.138

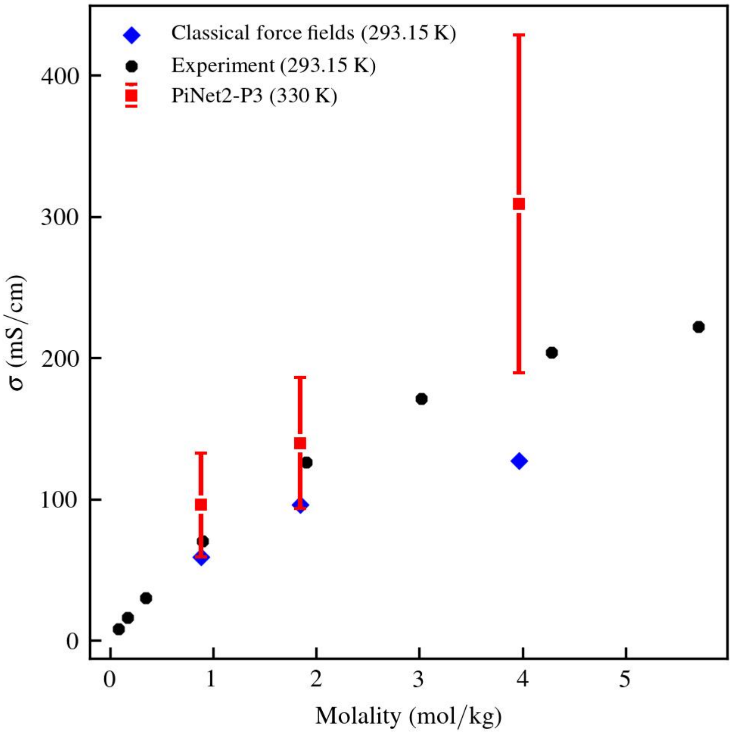

Figure S3: Ionic conductivities σ of NaCl aqueous solution systems shown in Table S12 calculated by Green-Kubo equation at 330 K in the NVT ensemble. The standard deviations were obtained from 7 independent models. The results from classical force fields[S12] and experiments[S13] are shown as references.

Computational setup, hyperparameters, the PiNet/PiNet2 comparison table for simulating protonic liquid liquids

The electronic structure calculations, including the preparation of the initial dataset and the labelling of MLP-sampled trajectories, are performed using the CP2K code.[S14] The molecularly optimized double-ζ basis set[S15] and the Goedecker-Teter-Hutter pseudopotentials.[S16–S18] and dispersion correction in the DFT-D3 scheme[S19] (in the case of revPBE functional) were applied. The self consistent field (SCF) computation was converged to the tolerance of 10^{-6} (`EPS_SCF`) using the DIIS optimizer, with a mixium of 100 inner loops and 10 outer loops. 5 (`NGRID`) multigrids were used, with a cutoff of 1200 Ry (`CUTOFF`) for the finest grid level, and a cutoff of 30 Ry (`REL_CUTOFF`) for the mapping of product Gaussians to the grids.

For training MLPs with PiNet and PiNet2-P3, the following hyperparameters have been used as specified in Tab. S13. Each MLP model was trained using the Adam optimizer[S5] with a learning rate of 0.0003, which decays by a factor of 0.994 every 10000 steps. The model was trained for 4×10^5 for the first generation; afterwards, an additional training of 2×10^5 steps will be applied whenever deviation of the MLP prediction from the CP2K label exceeds a given tolerance (Dashed lines in the Figure 7, Main Text).

Table S13: Hyperparameters used with the PiNet and the PiNet2-P3 architectures. See ref. S1 for a detailed description of the architecture

Architecture	Layer	Architecture	Parameter	Value
PiNet	PI	[16]×10	R_c	5.5 Å
	II	[16, 16, 16]	GC blocks	4
	PP	[16, 16, 16]	n_{basis}	10
	Output	[16, 1]	η	3.0 Å^{-2}
PiNet2-P3	PI	[16]×10	R_c	4.5 Å
	II	[16, 16, 16, 16]	GC blocks	5
	PP	[16, 16, 16, 16]	n_{basis}	10
	Output	[16, 1]	η	3.0 Å^{-2}

Table S14: The final valuation errors, and the generations of training and/or sampling loops to reach the convergence.

Model	RMSE_E (meV/atom)	RMSE_F (meV/Å)	Generation
PiNet/revPBE-D3	7.0	98.5	48
PiNet/SCAN	7.2	102.8	50
PiNet2-P3/revPBE-D3	10.0	34.2	4
PiNet2-P3/SCAN	11.9	40.7	9

Table S15: Self-diffusion coefficients D^{s} (10^{-2} Å^2 ps^{-1}) of active protons and acetate groups, the Green-Kubo conductivity $\sigma_{\text{G-K}}$ (mS cm^{-1}), the Nernst-Einstein deviation $\Delta = 1 - \sigma_{\text{G-K}}/\sigma_{\text{N-E}}$ obtained from MD simulations at 340 K using MLPs generated with PiNet2-P3 (denoted as "P3") and PiNet (denoted as "P1") and PiNNAcLe.

Model	$D^{\mathrm{s}}_{\mathrm{C_1Im}}$	$D^{\mathrm{s}}_{\mathrm{OAc}}$	$\sigma_{\mathrm{G-K}}$	Δ
P3/revPBE-D3	1.06(7)	0.97(9)	6(1)	0.89
P3/SCAN	3.7(1)	3.1(1)	14(6)	0.92
P1/revPBE-D3	1.00(8)	1.00(3)	7(2)	0.86
P1/SCAN	3.4(2)	2.69(2)	20(2)	0.87

Simulation setup of polarized graphene-oxide electrode

The MetalWalls code[S20,S21] was used as the MD engine for PiNNwall application, which was built for simulating electrochemical systems with Siepmann–Sprik-type models. A box size of 31.974 Å by 34.080 Å by 70.124 Å was used. 3D PBCs were applied with a real-space cutoff of 15.99 Å for the Ewald summation and the Lennard-Jones interactions. The electrode consists of seven graphene layers that have an interlayer spacing of 3.354 Å, resulting in 2912 carbon atoms, As a result, the electrolyte is 50 Å space along the z direction. Only the graphene layers at the interface with the electrolyte are functionalized with hydroxyl groups for the different surface coverages. The Lennard-Jones parameters of electrode atoms were taken from the OPLS-AA force field[S22] using Lorentz–Berthelot mixing rules to compute the cross pair parameters with the electrolyte. As for an electrolyte, we used an aqueous potassium chloride solution with a concentration of 1 mol/L. This results in 1901 water molecules and 35 ion pairs. Water was modeled with the TIP3P model[S23] and the ion models of aqueous K^+ and Cl^+ were taken from ref.[S24]

Each simulation consists in an equilibration run of 2 ns, followed by a production run of 10 ns. We used a time step of 2 fs in the NVT ensemble using with a relaxation time of 0.1 ps and a temperature of 300 K.

To predict the base charges of the functional groups, an approach using molecular analogues was used as detailed in ref.[S25] A PiNet2-dipole AC model trained on the QM7b dataset using the same hyperparameters as for the QM9 dataset as outlined above to predict the charges.

Table S16: Base charges from methanol as predicted from PiNet2 and implemented in the hydroxyl-terminated graphene oxide.

Element	Atomic charge (e)
O	-0.40170
H	0.30231
C	0.09939

Table S17: Base charges from the neutral carboxyl flake as predicted from PiNet2 and implemented in the neutral carboxyl-terminated graphene oxide.

Element	Atomic charge (e)
$O_{\text{double-bonded O}}$	-0.22630
O_{OH}	-0.24937
H_{OH}	0.34293
C	0.13274

Table S18: Base charges from the protonated carboxyl flake as predicted from PiNet2 and implemented in the protonated side of carboxyl-terminated graphene oxide.

Element	Atomic charge (e)
$O_{\text{double-bonded O}}$	-0.33754
$H_{\text{double-bonded O}}$	0.36940
O_{OH}	-0.27223
H_{OH}	0.33659
C	0.90378

Table S19: Base charges from the deprotonated carboxyl flake as predicted from PiNet2 and implemented in the deprotonated side of carboxyl-terminated graphene oxide.

Element	Atomic charge (e)
$O_{\text{double-bonded O}}$	-0.23816
O_{OH}	-0.23405
C	-0.52779

References

(S1) Shao, Y.; Hellström, M.; Mitev, P. D.; Knijff, L.; Zhang, C. PiNN: A Python Library for Building Atomic Neural Networks of Molecules and Materials. *J. Chem. Inf. Model.* **2020**, *60*, 1184–1193.

(S2) Schütt, K.; Unke, O.; Gastegger, M. Equivariant message passing for the prediction of tensorial properties and molecular spectra. Proceedings of the 38th International Conference on Machine Learning. 2021; pp 9377–9388.

(S3) Brehm, M.; Thomas, M.; Gehrke, S.; Kirchner, B. TRAVIS—A free analyzer for trajectories from molecular simulation. *J. Chem. Phys.* **2020**, *152*, 164105.

(S4) Thomas, M.; Brehm, M.; Fligg, R.; Vöhringer, P.; Kirchner, B. Computing vibrational spectra from ab initio molecular dynamics. *Phys. Chem. Chem. Phys.* **2013**, *15*, 6608–6622.

(S5) Kingma, D. P.; Ba, J. Adam: A Method for Stochastic Optimization. *arXiv:1412.6980 [cs]* **2017**, arXiv: 1412.6980.

(S6) Abadi, M.; Agarwal, A.; Barham, P.; Brevdo, E.; Chen, Z.; Citro, C.; Corrado, G. S.; Davis, A.; Dean, J.; Devin, M.; Ghemawat, S.; Goodfellow, I.; Harp, A.; Irving, G.; Isard, M.; Jia, Y.; Jozefowicz, R.; Kaiser, L.; Kudlur, M.; Levenberg, J.; Mane, D.; Monga, R.; Moore, S.; Murray, D.; Olah, C.; Schuster, M.; Shlens, J.; Steiner, B.; Sutskever, I.; Talwar, K.; Tucker, P.; Vanhoucke, V.; Vasudevan, V.; Viegas, F.;

Vinyals, O.; Warden, P.; Wattenberg, M.; Wicke, M.; Yu, Y.; Zheng, X. TensorFlow: Large-Scale Machine Learning on Heterogeneous Distributed Systems. 2016.

(S7) Pascanu, R.; Mikolov, T.; Bengio, Y. On the Difficulty of Training Recurrent Neural Networks. Proceedings of the 30th International Conference on International Conference on Machine Learning - Volume 28. 2013; pp 1310–1318.

(S8) Shao, Y.; Andersson, L.; Knijff, L.; Zhang, C. Finite-field coupling via learning the charge response kernel. *Electron. Struct.* **2022**, *4*, 014012.

(S9) Berendsen, H. J.; Postma, J. v.; Van Gunsteren, W. F.; DiNola, A.; Haak, J. R. Molecular dynamics with coupling to an external bath. *J. Chem. Phys.* **1984**, *81*, 3684–3690.

(S10) Larsen, A. H.; Mortensen, J. J.; Blomqvist, J.; Castelli, I. E.; Christensen, R.; Dułak, M.; Friis, J.; Groves, M. N.; Hammer, B.; Hargus, C., et al. The atomic simulation environment—a Python library for working with atoms. *J. Phys. Condens. Matter* **2017**, *29*, 273002.

(S11) Bussi, G.; Donadio, D.; Parrinello, M. Canonical sampling through velocity rescaling. *J. Chem. Phys.* **2007**, *126*.

(S12) Shao, Y.; Shigenobu, K.; Watanabe, M.; Zhang, C. Role of viscosity in deviations from the nernst–einstein relation. *J. Phys. Chem. B* **2020**, *124*, 4774–4780.

(S13) Weast, R. C. *CRC handbook of chemistry and physics*, 70th ed.; CRC Press Inc., Boca Raton, FL, 1989,; pp D–221.

(S14) Kühne, T. D.; Iannuzzi, M.; Ben, M. D.; Rybkin, V. V.; Seewald, P.; Stein, F.; Laino, T.; Khaliullin, R. Z.; Schütt, O.; Schiffmann, F.; Golze, D.; Wilhelm, J.; Chulkov, S.; Bani-Hashemian, M. H.; Weber, V.; Borštnik, U.; Taillefumier, M.;

Jakobovits, A. S.; Lazzaro, A.; Pabst, H.; Müller, T.; Schade, R.; Guidon, M.; Andermatt, S.; Holmberg, N.; Schenter, G. K.; Hehn, A.; Bussy, A.; Belleflamme, F.; Tabacchi, G.; Glöß, A.; Lass, M.; Bethune, I.; Mundy, C. J.; Plessl, C.; Watkins, M.; VandeVondele, J.; Krack, M.; Hutter, J. CP2K: An electronic structure and molecular dynamics software package - Quickstep: Efficient and accurate electronic structure calculations. *J. Chem. Phys.* **2020**, *152*, 194103.

(S15) VandeVondele, J.; Hutter, J. Gaussian basis sets for accurate calculations on molecular systems in gas and condensed phases. *J. Chem. Phys.* **2007**, *127*, 114105.

(S16) Goedecker, S.; Teter, M.; Hutter, J. Separable dual-space Gaussian pseudopotentials. *Phys. Rev. B* **1996**, *54*, 1703–1710.

(S17) Hartwigsen, C.; Goedecker, S.; Hutter, J. Relativistic separable dual-space Gaussian pseudopotentials from H to Rn. *Phys. Rev. B* **1998**, *58*, 3641–3662.

(S18) Krack, M. Pseudopotentials for H to Kr optimized for gradient-corrected exchange-correlation functionals. *Theor. Chem. Acc.* **2005**, *114*, 145–152.

(S19) Grimme, S.; Ehrlich, S.; Goerigk, L. Effect of the damping function in dispersion corrected density functional theory. *J. Comput. Chem.* **2011**, *32*, 1456–1465.

(S20) Marin-Laflèche, A.; Haefele, M.; Scalfi, L.; Coretti, A.; Dufils, T.; Jeanmairet, G.; Reed, S. K.; Serva, A.; Berthin, R.; Bacon, C.; Bonella, S.; Rotenberg, B.; Madden, P. A.; Salanne, M. MetalWalls: A classical molecular dynamics software dedicated to the simulation of electrochemical systems. *J. Open Source Softw.* **2020**, *5*, 2373.

(S21) Coretti, A.; Bacon, C.; Berthin, R.; Serva, A.; Scalfi, L.; Chubak, I.; Goloviznina, K.; Haefele, M.; Marin-Laflèche, A.; Rotenberg, B.; Bonella, S.; Salanne, M. MetalWalls: Simulating electrochemical interfaces between polarizable electrolytes and metallic electrodes. *J. Chem. Phys.* **2022**, *157*, 184801.

(S22) Jorgensen, W. L.; Maxwell, D. S.; Tirado-Rives, J. Development and testing of the OPLS all-atom force field on conformational energetics and properties of organic liquids. *J. Am. Chem. Soc.* **1996**, *118*, 11225–11236.

(S23) Jorgensen, W. L.; Chandrasekhar, J.; Madura, J. D.; Impey, R. W.; Klein, M. L. Comparison of simple potential functions for simulating liquid water. *J. Chem. Phys.* **1983**, *79*, 926–935.

(S24) Joung, I. S.; Cheatham, T. E. Determination of Alkali and Halide Monovalent Ion Parameters for Use in Explicitly Solvated Biomolecular Simulations. *J. Phys. Chem. B* **2008**, *112*, 9020–9041.

(S25) Dufils, T.; Knijff, L.; Shao, Y.; Zhang, C. PiNNwall: Heterogeneous Electrode Models from Integrating Machine Learning and Atomistic Simulation. *J. Chem. Theory Comput.* **2023**, *19*, 5199–5209.
